# Evaluating DNA Mixtures with Contributors from Different Populations Using Probabilistic Genotyping

**DOI:** 10.3390/genes14010040

**Published:** 2022-12-23

**Authors:** Maarten Kruijver, Hannah Kelly, Jo-Anne Bright, John Buckleton

**Affiliations:** 1 Institute of Environmental Science and Research, Auckland 1142, New Zealand; 2 Department of Statistics, University of Auckland, Auckland 1142, New Zealand

**Keywords:** probabilistic genotyping, DNA mixtures, population stratification, likelihood ratio

## Abstract

It is common practice to evaluate DNA profiling evidence with likelihood ratios using allele frequency estimates from a relevant population. When multiple populations may be relevant, a choice has to be made. For two-person mixtures without dropout, it has been reported that conservative estimates can be obtained by using the Person of Interest’s population with a θ value of 3%. More accurate estimates can be obtained by explicitly modelling different populations. One option is to present a minimum likelihood ratio across populations; another is to present a stratified likelihood ratio that incorporates a weighted average of likelihoods across multiple populations. For high template single source profiles, any difference between the methods is immaterial as far as conclusions are concerned. We revisit this issue in the context of potentially low-level and mixed samples where the contributors may originate from different populations and study likelihood ratio behaviour. We first present a method for evaluating DNA profiling evidence using probabilistic genotyping when the contributors may originate from different ethnic groups. In this method, likelihoods are weighted across a prior distribution that assigns sample donors to ethnic groups. The prior distribution can be constrained such that all sample donors are from the same ethnic group, or all permutations can be considered. A simulation study is used to determine the effect of either assumption on the likelihood ratio. The likelihood ratios are also compared to the minimum likelihood ratio across populations. We demonstrate that the common practise of taking a minimum likelihood ratio across populations is not always conservative when $F_{ST}=0$. Population stratification methods may also be non-conservative in some cases. When $F_{ST}>0$ is used in the likelihood ratio calculations, as is recommended, all compared approaches become conservative on average to varying degrees.

## 1. Introduction

DNA mixtures are routinely used to identify the donors of biological samples obtained from crime scenes [1,2,3]. If a Person of Interest (POI) is identified, a likelihood ratio (LR) statistic can be used to evaluate the evidence. Typically, when a POI is compared to a mixture, the standard hypothesis pair

(1) $$\begin{array}{cc} H_{1}:\;\mathrm{POI}\;\mathrm{and}\;N-1\;\mathrm{unrelated}\;\mathrm{persons}\;\mathrm{contributed}\;\mathrm{to}\;\mathrm{the}\;\mathrm{mixture},\\ H_{2}:\;N\;\mathrm{unrelated}\;\mathrm{persons}\;\mathrm{contributed}\;\mathrm{to}\;\mathrm{the}\;\mathrm{mixture}, & \; \end{array}$$

is used to evaluate support for the POI having contributed to the mixture. A population genetic model is needed to assign a likelihood ratio for a pair of hypotheses. It is common practice among users of probabilistic genotyping software to employ the Balding-Nichols model [4] with a parameter $F_{ST}>0$ (also called θ) chosen sufficiently large to obtain conservative estimates of the evidential weight. For $F_{ST}=0$ the Balding-Nichols model reduces to a population genetic model assuming Hardy Weinberg Equilibrium. If Linkage Equilibrium is also assumed, this model is also known as the Product Rule model [5]. Either way, allele frequencies obtained from a population study have to be used to assign allele probabilities in the likelihood ratio method. It is well known that a population study based on a representative sample from the relevant population needs to be used in the estimate of allele probabilities to avoid overstating the rarity of the observed variant alleles and consequently reporting an anti-conservative assessment of the weight of evidence [6]. Because match probabilities for unrelated persons not involved in a case for high quality profiles obtained using modern multiplexes are infinitesimally small regardless of the specific statistical evaluation method or population database [7], the problem of assigning the weight of evidence correctly may appear less important than before. On the other hand, the increased sensitivity of modern DNA profiling technology has dramatically increased the number of low-level and mixed samples that are routinely analysed which reinforces the need for accurate assessment of the weight of evidence because these samples are less informative [8]. Moreover, the use of expanded marker sets has increased the potential for population differentiation based on STRs [9] adding to the importance of using representative population databases and unbiased statistical methods.

In many jurisdictions, there are multiple populations that are potentially relevant when evaluating DNA evidence. For instance, the United States National Institute of Standards and Technology (NIST) laboratory has published allele frequency estimates for four population groups (African American, Caucasian, Hispanic and Asian) [10]. A more comprehensive set of estimates by the United States Federal Bureau of Investigation (FBI) [11] includes African Americans, Caucasians, Southwest Hispanics, Bahamians, Jamaicans, Trinidadians, Filipinos, and Chamorros. Depending on the geographical location and other case circumstances, more than one population may be relevant when evaluating the evidence in a particular case. For criminal casework, a pragmatic solution to the problem of choosing a relevant population could be to evaluate the likelihood ratio several times using different population parameters and reporting the minimum likelihood ratio statistic across the populations as a conservative estimate of the weight of evidence. This is established practice in some forensic laboratories. Further, this approach is one of the two options recommended by the SWGDAM Ad Hoc Working Group on Genotyping Results Reported as Likelihood Ratios [12] and has been studied in the context of relationship testing [13] but not in the context of DNA mixture interpretation. Note that this approach does not take into account the possibility that different donors originate from different populations and may not be conservative if this is the case. Alternatively, the working group states: “*a likelihood ratio that combines information from the different population groups (e.g., weighted averages of the likelihoods) may be used in lieu of the single lowest value*”. The working group agrees with the DNA Commission of the International Society for Forensic Genetics (ISFG), who write that “*methods for stratifying the multiple evidential weights that have been obtained using the separate ethnic databases into a single value*” are a “*very elegant solution*” [14]. In practice, such a method is likely to be a population stratified likelihood ratio that, in one way or another, models the different populations with some prior probabilities in the likelihood calculations. The prior probabilities can be based on census proportions or perhaps on case-specific background information. We are aware of this practice being adopted in some labs in Australasia and the United States of America.

Despite the apparent consensus that the use of population stratified likelihood ratios would be beneficial, little is published about how such likelihood ratios should be computed exactly and their behaviour with regards to different modelling choices appears to be poorly understood. For single source profiles, it has been shown that a population stratified likelihood ratio can be obtained from a weighted harmonic mean of the likelihood ratios for individual populations [15]. Specifically, if $\pi_{e}$ determines the probability that the donor of a sample originates from population *e* and ${\mathrm{LR}}_{e}$ is calculated for population *e*, then a stratified likelihood ratio can be computed as:

$$\frac{1}{\mathrm{LR}}=\sum_{e}\frac{1}{{\mathrm{LR}}_{e}}\pi_{e}.$$

When the DNA profile is mixed, a population stratified likelihood ratio can in general not be obtained from the separate likelihood ratios assuming different populations [15]. A further complication is that the different contributors to a mixture may originate from different populations. This can be taken into account in the likelihood ratio calculations. As far as we are aware, there are no published studies that evaluate the different modelling choices that can be made. For the evaluation of mixed profiles, the STRmix^TM^ software implements a population stratified likelihood ratio that uses weighted averages of the likelihoods across population groups in the numerator and the denominator:

(2) $${\mathrm{LR}}_{\mathrm{STRmix}}=\frac{\sum_{e}\pi_{e}P\left(O|H_{1},G_{p},e\right)}{\sum_{e}\pi_{e}P\left(O|H_{2},G_{p},e\right)}=\frac{\sum_{e}\pi_{e}\sum_{s}P\left(O|s\right)P\left(s|H_{1},G_{p},e\right)}{\sum_{e}\pi_{e}\sum_{s}P\left(O|s\right)P\left(s|H_{2},G_{p},e\right)},$$

where *O* is the observed mixture, *e* is a population, *s* denotes an ordered genotype set, P(O|s) is the weight of a genotype set and $G_{p}$ is the genotype of a POI. Thus, the numerator and denominator likelihoods are computed in each population and weighted by their prior probabilities. When $F_{ST}>0$, a sub-population correction is applied taking into account the POI’s alleles for each population. The behaviour of this likelihood ratio has not been studied before. We make one initial observation. From (2), it can be seen that the numerator is fixed (across populations) as long as the sample is single source and there are no drop-in alleles so that the numerator equals the weight of the genotype in the deconvolution. In [15], it was shown the likelihood ratio simplifies to the harmonic mean in this case.

The DBLR^TM^ software [16] version 1.2 implements a likelihood ratio framework for the evaluation of propositions involving one or more samples where contributors may be related according to a pedigree [17]. The comparison of a POI to a mixture can be formulated within this framework. Our intention is to develop an updated version of the software that is able to flexibly model population stratification in this general framework so that the population of each founder in the pedigree can be modelled separately. As a first step towards this development, we have implemented population stratification in a probabilistic way for pedigrees comprising founders only. The population from which each person originates is modelled using a prior distribution. The joint prior distribution of the populations for all persons can be constrained such that all sample donors are from the same ethnic group, or all permutations of populations can be considered.

The aim of the current work is to employ a simulation study to investigate the effect of different modelling choices when computing population stratified likelihood ratios. Specifically, we introduce and study two approaches within the proposed DBLR^TM^ framework. In the first approach, which we call *simple stratification*, each person is assumed to originate from the same population. In the second approach, which we call *full stratification*, the population of all persons is considered to be independent *a priori*. Hence, all permutations of assignments of persons to populations are considered. These two approaches are compared to the method that is implemented in the STRmix^TM^ software (2) and the pragmatic option of considering a minimum across population-specific likelihood ratios.

## 2. Methods

### 2.1. Likelihood Ratio Framework

The likelihood ratio framework presented in [17] is briefly summarised here. We have observed M≥1 evidentiary samples which we denote $O_{1},\ldots ,O_{M}$. Sample $O_{i}$ is assumed to have been interpreted using probabilistic genotyping software assuming $N_{i}\geq 1$ contributors so that the weights of genotype sets are determined at every locus. We seek to evaluate a likelihood ratio for two hypotheses $H_{1}$ and $H_{2}$ about the contributions of persons to samples. Formally, a hypothesis $H_{j}$ comprises a pedigree $P_{j}$ and a set of links $L_{j}$ from persons to samples. A link from a person to a sample may be specific to a contributor position or may span multiple contributor positions if the sample is mixed. In the current work, we will restrict the pedigrees to contain unrelated persons only.

We extend the framework by introducing a random variable $E=(E_{1},\ldots ,E_{m})$ that models the populations from which the *m* (unrelated) persons in a pedigree originate. When modelling r≥1 populations, the random variables $E_{i}$ take values in $e_{1},\ldots ,e_{r}$. In general, we need to specify a prior probability distribution for *E*. We consider two options. The first option is to restrict all persons to originate from the same population and to set $P\left(E_{1}=e,E_{2}=e,\ldots ,E_{m}=e\right)=\pi_{e}$. We will refer to this first option as *simple* stratified. A second option, which we full refer to as *fully* stratified, is to consider all permutations of populations and assume independence of population between persons. That is, $P\left(E_{1}=e_{1},E_{2}=e_{2},\ldots ,E_{m}=e_{m}\right)=\pi_{e_{1}}\pi_{e_{2}}\cdots \pi_{e_{m}}$. These two parametrisation of the prior distribution of *E* allow for a parsimonious model that can be informed by the prior population proportions. Although a more general specification is possible, it is unlikely that the prior probabilities can be meaningfully assigned in practice.

A hypothesis $H_{i}$ as just defined is composite in two senses. First, the links from persons to samples may involve multiple contributor positions as explained in [17]. For instance, a person may be assumed to contribute to a two-person mixture in either contributor position (major or minor). To numerically evaluate the likelihood of a composite hypothesis, it first needs to be broken down into simple hypotheses. We employ the commonly applied assumption that each assignment of links to specific contributor positions has equal prior probability. Secondly, the hypothesis is composite in the sense that it is paired with a prior probability distribution of assignments of persons to populations.

Specifically, we partition a composite hypothesis $H_{i}$ into simple hypotheses $H_{ij}$ with prior probabilities $\alpha_{ij}$ such that $\sum_{j}\alpha_{ij}=1$. Such a simple hypothesis comprises a pedigree, a set of links from persons to samples at specific contributor positions and the population from which each person originates. The likelihood of $H_{i}$ may then be evaluated as:

$$\begin{array}{cc} P(O_{1},\ldots ,O_{M}|H_{i}) & =\sum_{j}\alpha_{ij}P\left(O_{1},\ldots ,O_{M}|H_{ij}\right)\\ \; & =\sum_{j}\alpha_{ij}\sum_{g_{1},\ldots ,g_{m}}P\left(g_{1},\ldots ,g_{m}|P_{ij},e_{ij}\right)\prod_{k=1}^{M}P\left(O_{k}|g_{1},\ldots ,g_{m},L_{ij}\right). \end{array}$$

This is a generalisation incorporating population assignments for an expression derived previously ([17], Section 2.1). The outer sum considers all simple hypotheses, now incorporating population assignments and links from persons to samples at specific contributor positions. The inner sum is as defined before in [17]. That is, all possible genotypes are considered for pedigree members and the probability of the genotypes are computed using a pedigree algorithm. The product $\prod_{k=1}^{M}P\left(O_{k}|g_{1},\ldots ,g_{m},L_{ij}\right)$ is computed from the sample deconvolutions.

### 2.2. Likelihood Ratio for Comparison of a POI to a Single Sample

We will derive simple expressions for the likelihood ratio calculations outlined in the special case that a POI is compared to a single mixture. This will facilitate a comparison of the different approaches. The starting point in the stratified likelihood ratio calculation in STRmix^TM^ (2) is the probability of the observed mixture profile conditional on the genotype of a POI. The DBLR^TM^ approach, on the other hand, includes the probability of all genotypes in both the numerator and denominator likelihoods. Below, we derive expressions for the DBLR^TM^ likelihood ratio for the specific case of the comparison of a POI to a two-person mixture to facilitate comparison with the STRmix^TM^ approach.

#### 2.2.1. Simple Stratification

The simple stratification method considers all persons to be from the same population, regardless of whether a person is a mixture contributor or the POI. The likelihood of a hypothesis $H_{j}$ (j=1,2) can be written as a sum where each term considers a population:

$$\begin{array}{cc} P(O|H_{j},G_{p}) & =\sum_{e}P\left(O|H_{j},G_{p},e\right)P\left(e|H_{j},G_{p}\right)=\sum_{e}P\left(O|H_{j},G_{p},e\right)P\left(e|G_{p}\right). \end{array}$$

The term $P(O|H_{j},G_{p},e)$ is the likelihood of the observed mixture assuming population *e* and conditional on the POI’s genotype $\left(G_{p}\right)$. The term $P(e|G_{p})$ is the *posterior* probability that all persons are from population *e* after observing the POI’s genotype. We denote this probability as $p_{e|G_{p}}:=P\left(e|G_{p}\right)$ and compute it by applying Bayes’ rule:

$$p_{e|G_{p}}=\frac{\pi_{e}P\left(G_{p}|e\right)}{\sum_{e}\pi_{e}P\left(G_{p}|e\right)}.$$

Hence, the simple stratified likelihood ratio is evaluated as:

$$\begin{array}{cc} {\mathrm{LR}}_{\mathrm{simple}} & =\frac{P(O|H_{1},G_{p})}{P(O|H_{2},G_{p})}\\ \; & =\frac{\sum_{e}p_{e|G_{p}}P\left(O|H_{1},G_{p},e\right)}{\sum_{e}p_{e|G_{p}}P\left(O|H_{2},G_{p},e\right)}\\ \; & =\frac{\sum_{e}p_{e|G_{p}}\sum_{s}P\left(O|s\right)P\left(s|H_{1},G_{p},e\right)}{\sum_{e}p_{e|G_{p}}\sum_{s}P\left(O|s\right)P\left(s|H_{2},G_{p},e\right)} \end{array}$$

Comparing this expression to (2), it is seen that the prior probabilities in (2) are replaced by posterior probabilities. These posterior probabilities appear as a result of the assumption of perfect dependence of the populations of all relevant persons: if the POI is from a certain population, then all persons are assumed to be from this population. Hence, conditional on the POI’s observed genotype $\left(G_{p}\right)$, the posterior probability that all persons are from population *e* is equal to the posterior probability that the POI is from population *e*. As will be demonstrated in example calculations below, if no sub-population correction is applied, i.e., $F_{ST}=0$, then the denominator can be simplified because the genotypes of the mixture donors and the POI are independent.

#### 2.2.2. Full Stratification

The fully stratified likelihood considers all permutations of population assignments. If we write the likelihoods conditional on the POI’s genotype, then it is helpful to first decompose $H_{1}$ into sub-hypotheses $H_{11},H_{12},\ldots ,H_{1N}$ which state that the POI is donor 1 up to *N* respectively, where *N* is the number of contributors used in the deconvolution and then consider the population assignments.

$$\begin{array}{cc} {\mathrm{LR}}_{fully-stratified}= & \frac{P(O|H_{1},G_{p})}{P(O|H_{2},G_{p})}\\ = & \frac{\frac{1}{N}\left(P\left(O|H_{11},G_{p}\right)+P\left(O|H_{12},G_{p}\right)\right)+\cdots +P\left(O|H_{1N},G_{p}\right)}{P(O|H_{2},G_{p})} \end{array}$$

Each term in the numerator requires a sum over all permutations of population assignments

$$\begin{array}{cc} P(O|H_{1i},G_{p}) & =\sum_{e_{1},e_{2},\ldots ,e_{N}}P\left(e_{1},e_{2},\ldots ,e_{N}|H_{1i},G_{p}\right)P\left(O|H_{1i},G_{p},e_{1},e_{2},\ldots ,e_{N}\right)\\ \; & =\sum_{e_{1},e_{2},\ldots ,e_{N}}\left(p_{e_{i}|G_{p}}\prod_{j\neq i}\pi_{e_{j}}\right)P\left(O|H_{1i},G_{p},e_{1},e_{2},\ldots ,e_{N}\right) \end{array}$$

In sub-hypothesis $H_{1i}$, the POI is contributor *i* to the mixture, so the population of contributor *i* is the same as the POI’s. Each of the terms $P(O|H_{1i},G_{p},e_{1},e_{2},\ldots ,e_{N})$ is computed by explicit summation over all genotype combinations. If $F_{ST}>0$ then the Balding-Nichols approach is used for allele probabilities with each set of donors from the same population. The denominator of the likelihood ratio is also obtained by explicit summation over all population assignments.

$$\begin{array}{cc} P(O|H_{2},G_{p})= & \sum_{e_{1},e_{2},\ldots ,e_{N},e_{P}}\left(P\left(e_{1},e_{2},\ldots ,e_{N},e_{p}|H_{2},G_{p}\right)P\left(O|H_{2},G_{p},e_{1},e_{2},\ldots ,e_{N},e_{P}\right)\right)\\ = & \sum_{e_{1},e_{2},\ldots ,e_{N},e_{P}}(\pi_{e_{1}}\pi_{e_{2}}\cdots \pi_{e_{N}}p_{e_{p}|G_{p}}\sum_{s}P\left(O|s\right)P\left(s|H_{2},G_{p},e_{1},e_{2},\ldots ,e_{N},e_{p}\right)) \end{array}$$

If $F_{ST}=0$ this expression can be simplified as will be shown in the example calculations below.

#### 2.2.3. Example Calculations

**Figure 1:**
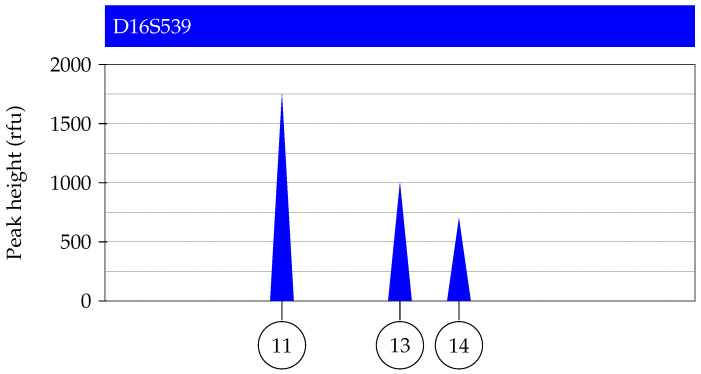
Constructed two-person mixture at a single locus.

To illustrate the differences between the likelihood ratio methods, we construct a one-locus example and demonstrate the explicit calculation of the likelihood ratios. Figure 1 shows a mock electropherogram for one locus of a two-person mixture; note that stutter peaks are not included for simplicity. Table 1 shows the corresponding deconvolution, i.e., the genotype sets and their corresponding weights. We assume there are two populations which we will label *A* and *B*. Table 2 shows (fictitious) allele frequencies in the two populations. We compare a POI with genotype (13/14) to the mixture and evaluate likelihood ratios for the hypothesis pair (1). For simplicity we use $F_{ST}=0$.

**Population specific likelihood ratios**. Using allele frequencies from population *A* only, we write ${\mathrm{LR}}_{A}=P_{A}\left(O|H_{1},G_{p}\right)/P_{A}\left(O|H_{2},G_{p}\right)$ where the subscript *A* emphasises the population that is used in the calculations. Because this is a two-person mixture and we evaluate the sub-source likelihood ratio, we consider two sub-hypotheses $H_{11}$ and $H_{12}$ stating respectively that the POI is the first or the second contributor. These sub-hypotheses are assumed to have equal prior probability. In this example, the POI genotype is (13/14). Hence, $P_{A}\left(O|H_{1},G_{p}\right)=\frac{1}{2}\left(P_{A}\left(O|H_{11,G_{p}}\right)+P_{A}\left(O|H_{12,G_{p}}\right)\right)$. There is only a single matching genotype combination, so we obtain $P_{A}\left(O|H_{11,G_{p}}\right)={\left(f_{11}^{A}\right)}^{2}\times 0.3$ and $P_{A}\left(O|H_{12,G_{p}}\right)={\left(f_{11}^{A}\right)}^{2}\times 0.2$ which yields $P_{A}\left(O|H_{1,G_{p}}\right)={\left(f_{11}^{A}\right)}^{2}\times 0.25$. To evaluate $P_{A}\left(O|H_{2},G_{p}\right)$, we sum over the genotype combinations:

$$\begin{array}{cc} P_{A}\left(O|H_{2},G_{p}\right)= & \sum_{s}P_{A}\left(s|H_{2},G_{p}\right)P\left(O|H_{2},G_{p},s\right)\\ = & \sum_{s}P_{A}\left(s|H_{2}\right)w_{s}\\ = & 2f_{13}^{A}f_{14}^{A}{\left(f_{11}^{A}\right)}^{2}\times 0.3+4f_{11}^{A}f_{14}^{A}f_{11}^{A}f_{13}^{A}\times 0.27+\\ \; & 4f_{11}^{A}f_{13}^{A}f_{11}^{A}f_{14}^{4}\times 0.23+2{\left(f_{11}^{A}\right)}^{2}f_{13}^{A}f_{14}^{A}\times 0.2\\ = & 3{\left(f_{11}^{A}\right)}^{2}f_{13}^{A}f_{14}^{A} \end{array}$$

We obtain the likelihood ratio in population *A* as ${\mathrm{LR}}_{A}=\frac{1}{12f_{13}^{A}f_{14}^{A}}\approx 2.78$ using frequencies in Table 2. Analogously, we obtain ${\mathrm{LR}}_{B}=\frac{1}{12f_{13}^{B}f_{14}^{B}}\approx 1.39$.

**Simple stratification**. To evaluate the likelihood ratio using simple stratification we need the posterior probabilities that the POI’s genotype originates from population *A* or *B*. The probability of the POI’s genotype in population *A* is $P_{A}\left(G_{p}\right)=2f_{13}^{A}f_{14}^{A}=0.06$ and the probability of the POI’s genotype in population *B* is $P_{B}\left(G_{p}\right)=2f_{13}^{B}f_{14}^{B}=0.12$. Hence, the posterior probability that the POI’s genotype originates from population *A* equals $p_{A|G_{p}}=\frac{\frac{1}{2}0.06}{\frac{1}{2}0.06+\frac{1}{2}0.12}=\frac{1}{3}$; and $p_{B|G_{p}}=\frac{2}{3}$. Next, we need to evaluate

$$\begin{array}{cc} {\mathrm{LR}}_{\mathrm{simple}} & =\frac{P(O|H_{1},G_{p})}{P(O|H_{2},G_{p})}\\ \; & =\frac{\sum_{e}p_{e|G_{p}}P\left(O|H_{1},G_{p},e\right)}{\sum_{e}p_{e|G_{p}}P\left(O|H_{2},G_{p},e\right)}\\ \; & =\frac{\frac{1}{3}P_{A}\left(O|H_{1},G_{p}\right)+\frac{2}{3}P_{B}\left(O|H_{1},G_{p}\right)}{\frac{1}{3}P_{A}\left(O|H_{2}\right)+\frac{2}{3}P_{B}\left(O|H_{2}\right)}. \end{array}$$

Previous calculations gave $P_{A}\left(O|H_{1},G_{p}\right)=\frac{1}{4}{\left(f_{11}^{A}\right)}^{2}$, $P_{B}\left(O|H_{1},G_{p}\right)=\frac{1}{4}{\left(f_{11}^{B}\right)}^{2}$, $P_{A}\left(O|H_{2}\right)=3{\left(f_{11}^{A}\right)}^{2}f_{13}^{A}f_{14}^{A}$ and $P_{B}\left(O|H_{2}\right)=3{\left(f_{11}^{B}\right)}^{2}f_{13}^{B}f_{14}^{B}$. We obtain

$$\begin{array}{cc} {\mathrm{LR}}_{\mathrm{simple}} & =\frac{\frac{1}{12}{\left(f_{11}^{A}\right)}^{2}+\frac{1}{6}{\left(f_{11}^{B}\right)}^{2}}{{\left(f_{11}^{A}\right)}^{2}f_{13}^{A}f_{14}^{A}+2{\left(f_{11}^{B}\right)}^{2}f_{13}^{B}f_{14}^{b}}=\frac{1075}{612}\approx 1.76. \end{array}$$

**Full stratification**. The full stratification method considers all permutations of population assignments. Under $H_{1}$, this means that the populations of the two mixture donors $\left(E_{1},E_{2}\right)$ can take the values (A,A), (A,B), (B,A) or (B,B). Under $H_{2}$, the populations of the two mixture donors and the POI $\left(E_{,}E_{2},E_{p}\right)$ can take eight values. We first decompose the sub-source hypothesis $H_{1}$ into $H_{11}$ and $H_{12}$ and then consider different population assignments in the calculation.

$$\begin{array}{cc} {\mathrm{LR}}_{fully-stratified}= & \frac{P(O|H_{1},G_{p})}{P(O|H_{2},G_{p})}=\frac{\frac{1}{2}\left(P\left(O|H_{11},G_{p}\right)+P\left(O|H_{12},G_{p}\right)\right)}{P(O|H_{2},G_{p})} \end{array}$$

Each term is computed separately.

$$\begin{array}{cc} P(O|H_{11},G_{p})= & \sum_{e_{1},e_{2}}P\left(E_{1}=e_{1},E_{2}=e_{2}|H_{11},G_{p}\right)P\left(O|H_{11},G_{p},e_{1},e_{2}\right)\\ = & \sum_{e_{1},e_{2}}p_{e_{1}|G_{p}}\pi_{e_{2}}P\left(O|H_{11},G_{p},e_{1},e_{2}\right) \end{array}$$

Recall that $p_{A|G_{p}}=\frac{1}{3}$ and $p_{B|G_{p}}=\frac{2}{3}$. Hence,

$$\begin{array}{cc} P(O|H_{11},G_{p})= & \frac{1}{3}\frac{1}{2}P_{AA}\left(O|H_{11},G_{p}\right)+\frac{1}{3}\frac{1}{2}P_{AB}\left(O|H_{11},G_{p}\right)+\\ \; & \frac{2}{3}\frac{1}{2}P_{BA}\left(O|H_{11},G_{p}\right)+\frac{2}{3}\frac{1}{2}P_{BB}\left(O|H_{11},G_{p}\right)\\ = & \frac{1}{6}0.3{\left(f_{11}^{A}\right)}^{2}+\frac{1}{6}0.3{\left(f_{11}^{B}\right)}^{2}+\frac{1}{3}0.3{\left(f_{11}^{A}\right)}^{2}+\frac{1}{3}0.3{\left(f_{11}^{B}\right)}^{2}\\ = & 0.15\left({\left(f_{11}^{A}\right)}^{2}+{\left(f_{11}^{B}\right)}^{2}\right) \end{array}$$

Similar calculations show that $P\left(O|H_{12},G_{p}\right)=0.1\left({\left(f_{11}^{A}\right)}^{2}+{\left(f_{11}^{B}\right)}^{2}\right)$. To compute $P(O|H_{2},G_{p})$, we do not need to explicitly sum over the eight possible population assignments because the genotypes of the mixture donors and the POI are independent given $F_{ST}=0$. Hence,

$$\begin{array}{cc} P(O|H_{2},G_{p})= & P(O|H_{2})\\ = & \pi_{A}\pi_{A}P_{AA}\left(O|H_{2}\right)+\pi_{A}\pi_{B}P_{AB}\left(O|H_{2}\right)+\\ \; & \pi_{B}\pi_{A}P_{BA}\left(O|H_{2}\right)+\pi_{B}\pi_{B}P_{BB}\left(O|H_{2}\right)\\ = & \frac{1}{4}3{\left(f_{11}^{A}\right)}^{2}f_{13}^{A}f_{14}^{A}+\frac{1}{4}(0.3\times 2\times f_{13}^{A}f_{14}^{A}{\left(f_{11}^{B}\right)}^{2}+0.27\times 4f_{11}^{A}f_{14}^{A}f_{11}^{B}f_{13}^{B}+\\ \; & 0.23\times 4\times f_{11}^{A}f_{13}^{A}f_{11}^{B}f_{14}^{B}+0.2\times 2\times {\left(f_{11}^{A}\right)}^{2}f_{13}^{B}f_{14}^{B})+\\ \; & \frac{1}{4}(0.3\times 2\times f_{13}^{B}f_{14}^{B}{\left(f_{11}^{A}\right)}^{2}+0.27\times 4f_{11}^{B}f_{14}^{B}f_{11}^{A}f_{13}^{A}+\\ \; & 0.23\times 4\times f_{11}^{B}f_{13}^{B}f_{11}^{A}f_{14}^{A}+0.2\times 2\times {\left(f_{11}^{B}\right)}^{2}f_{13}^{A}f_{14}^{A})+\\ \; & \frac{1}{4}3{\left(f_{11}^{B}\right)}^{2}f_{13}^{B}f_{14}^{B}\\ = & \frac{321}{8000}=0.040125 \end{array}$$

Combining the above, we arrive at ${\mathrm{LR}}_{fully-stratified}=\frac{0.125(0.6^{2}+0.5^{2})}{0.040125}\approx 1.90$.

**STRmix^TM^ stratification**. The population stratification method implemented in STRmix^TM^ considers a weighted average of the likelihoods in each population. For our example, we obtain

$$\begin{array}{cc} {\mathrm{LR}}_{\mathrm{STRmix}}= & \frac{\sum_{e}\pi_{e}P\left(O|H_{1},G_{p},e\right)}{\sum_{e}\pi_{e}P\left(O|H_{2},G_{p},e\right)}=\frac{\pi_{A}P_{A}\left(O|H_{1},G_{p}\right)+\pi_{B}P_{B}\left(O|H_{1},G_{p}\right)}{\pi_{A}P_{A}\left(O|H_{2}\right)+\pi_{B}P_{B}\left(O|H_{2}\right)}\\ = & \frac{\frac{1}{2}0.25{\left(f_{11}^{A}\right)}^{2}+\frac{1}{2}0.25{\left(f_{11}^{B}\right)}^{2}}{\frac{1}{2}3{\left(f_{11}^{A}\right)}^{2}f_{13}^{A}f_{14}^{A}+\frac{1}{2}3{\left(f_{11}^{B}\right)}^{2}f_{13}^{B}f_{14}^{B}}\approx 1.97 \end{array}$$

### 2.3. Simulation Study

In total, 250 two-person DNA mixtures were simulated for a GlobalFiler^TM^ kit using the simDNAmixtures [18,19] package for R. The template parameter of the major contributor was kept fixed at 500 rfu, while the minor contributor had a template parameter sampled uniformly between 25 (a third of the analytical threshold) and 125 rfu. The low template values for the minor contributor ensure that a large proportion of the samples exhibit severe dropout. The genotypes of the two contributors were sampled according to NIST allele frequencies [10], one from the African American sample and the other from the Caucasian sample. It was randomised which one was the major and which one was the minor contributor. All samples were interpreted using STRmix^TM^ version 2.9.

The goal of the simulation study was to compare likelihood ratios obtained using different ways of population stratification, and no population stratification, with the likelihood ratios computed using ground truth known populations. Likelihood ratios were assigned for the two known contributors to each of the 250 samples (500 likelihood ratios). Table 3 provides an overview of the permutations of parameters and implementations (DBLR^TM^ and STRmix^TM^) that were used in the calculations. The effect of not using population stratification was investigated first by evaluating likelihood ratios using African American allele frequencies only and Caucasian allele frequencies only using both DBLR^TM^ and STRmix^TM^. Next, the different ways of population stratification were applied. All likelihood ratio calculations were first done with $F_{ST}=0$ and then repeated with $F_{ST}=0.01$. The simulations were not repeated, i.e., the mixture donors were simulated according to a population genetic model with $F_{ST}=0$ in both cases.

The methods for population stratification discussed here are not restricted to mixtures of two contributors only. For example, if a likelihood ratio is evaluated involving a mixture of three contributors considering populations *A* and *B*, then eight permutations of population assignments are possible for the three mixture contributors. We have not involved three-person or higher order mixtures in the simulation study to keep the results concise.

### 2.4. Contributors from the Same Population

Although the main point of the current work is to investigate how the different likelihood ratio methods perform when the mixture contributors originate from different populations, we also perform one experiment where the contributors originate from the same population. Specifically, we repeat the simulation study described above for 250 mixed profiles where the contributors are simulated from the same populations.

## 3. Results

### 3.1. The Case of $F_{ST}=0$

**Figure 2:**
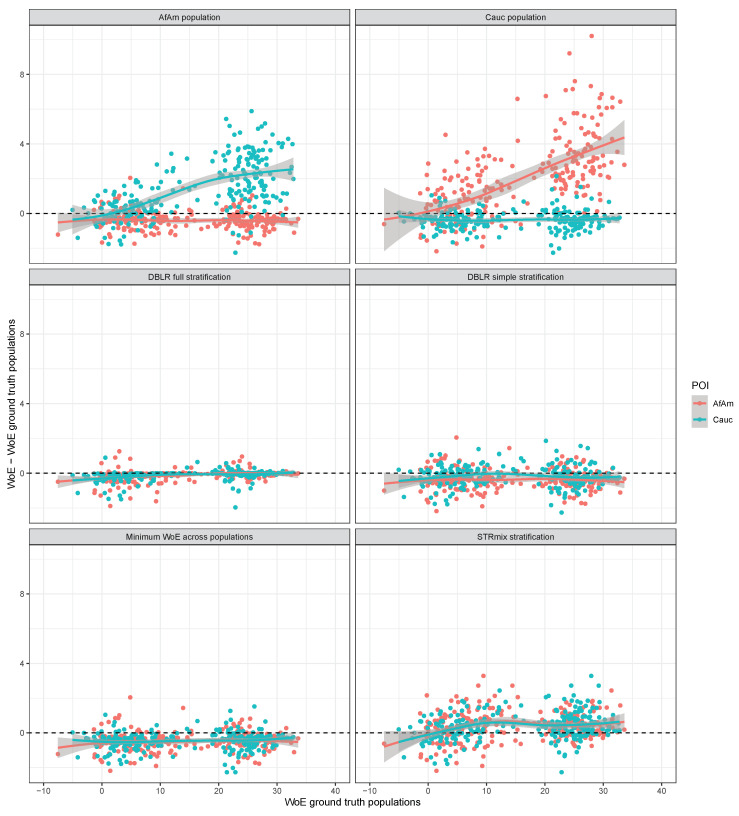
WoE difference the compared methods and the WoE evaluated the ground truth populations with $F_{ST}=0$ for the 500 donors to the 250 simulated two-person mixtures where the contributors originate from different populations.

We first discuss the effects of using the various methods listed in Table 3 for the case $F_{ST}=0$. Figure 2 compares the weight of evidence (WoE or $\log_{10}\mathrm{LR}$) for the true donors in the simulated mixtures obtained using each of the compared methods with the WoE obtained using the ground truth populations. Specifically, the vertical axis shows the difference between the WoE obtained using a particular method and the WoE obtained using the ground truth populations while the latter is shown on the horizontal axis. A positive difference means that the method is anti-conservative. First, we note that using a single population which does not correspond to the POI’s population may lead to an inflated WoE when the two mixture donors are from different populations. The top-left panel shows that for Caucasian POIs the WoE is up to six bans higher (i.e., the LR is up to six orders of magnitude higher) when African American allele frequencies are used than when the ground truth of mixed populations is used. Similarly, the top-right panel shows that for African American POIs the WoE is up to 9 bans higher when Caucasian allele frequencies are used in comparison to the ground truth populations. In both cases, however, using only the population of the POI is on average conservative. For African American POIs the median WoE difference when using only the African American population is −0.38; the median WoE difference for Caucasian POIs is −0.34. This can also be seen from the plots in which the trendlines sit slightly below zero.

The three methods for stratified likelihood ratios are compared next. The simple stratification method implemented in DBLR^TM^ assumes that all mixture donors are either from the one or from the other population which does not correspond to the ground truth. For some POIs this leads to an inflated WoE with the largest WoE difference being about two bans which may be a meaningful difference in some circumstances. The median WoE difference for the simple stratification method is −0.30 with a standard deviation of 0.57. The full stratification method implemented in DBLR^TM^ does take into account the possibility that mixture donors originate from different populations. The median WoE difference for the full stratification method is −0.03 with a standard deviation of 0.32. Hence, the full stratification method is more accurate and less biased than the simple stratification method in these simulations. The STRmix^TM^ stratification method (bottom-right panel) employs a weighted average of likelihoods in the numerator and denominator of the likelihood ratio. This method is on average slightly anti-conservative with a median WoE difference of 0.31 and a standard deviation of 0.82. For all simulated POIs the WoE difference was less than four bans.

Finally, we discuss the results for the approach of taking the minimum WoE across the two populations. Although this leads to the most conservative WoE across the two populations that are considered, the calculations do not take into account the possibility that different donors originate from different populations. Therefore the minimum WoE across populations can be larger than the WoE obtained using ground truth populations and may be anti-conservative. The bottom-left panel of Figure 2 shows the WoE difference for this method. The trendlines sit slightly below the horizontal axis which indicates that this method is on average conservative. The median WoE difference is −0.42 with a standard deviation of 0.52. For 13% of the simulated POIs this method is anti-conservative with respect to the WoE obtained using the ground truth populations (WoE difference greater than zero).

### 3.2. The Case of $F_{ST}=0.01$

**Figure 3:**
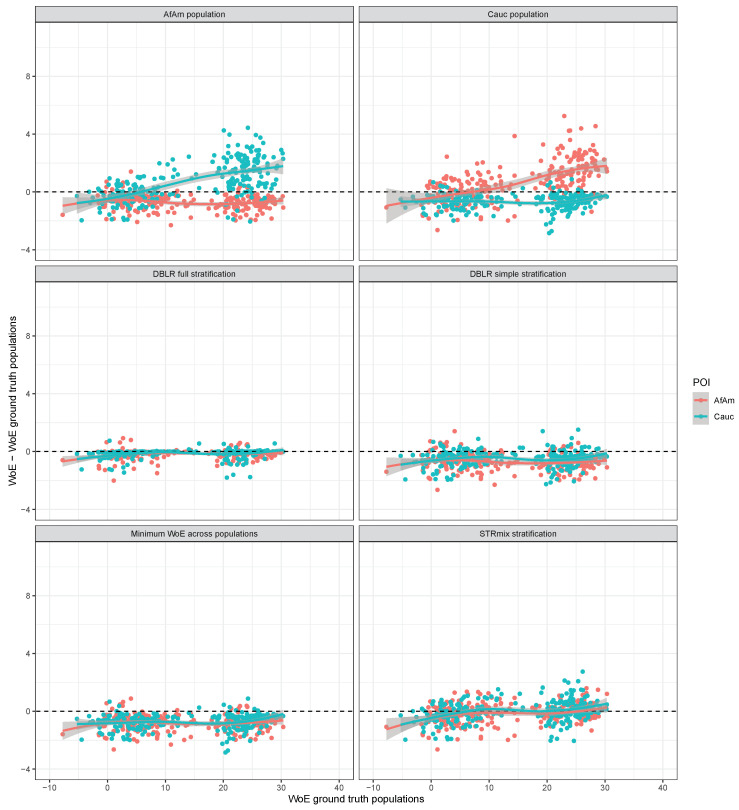
WoE difference between compared methods and the WoE evaluated the ground truth populations with $F_{ST}=0.01$ for the 500 donors to the 250 simulated two-person mixtures with contributors from different populations.

All likelihood ratio calculations comparing the POIs to the simulated mixtures were repeated with $F_{ST}=0.01$. We compare the results to the ones presented for $F_{ST}=0$ in the previous section. Figure 3 is the equivalent of Figure 2 with $F_{ST}=0.01$ instead of $F_{ST}=0$. The two top panes in Figure 3 show that using a single population in the likelihood ratio calculation that is not the POI’s population inflates the WoE for most POIs in comparison to the WoE evaluated using the ground truth populations. The extent to which the WoE is inflated is smaller than when $F_{ST}=0$ is used in the likelihood ratio calculations. Using only the POI’s population is conservative, as was the case with $F_{ST}=0$. Specifically, the WoE difference for African American POIs has a median of −0.61 and a standard deviation of 0.53; the WoE difference for Caucasian POIs has a median of −0.53 with a standard deviation of 0.52.

The three stratification methods are affected differently by the use of a sub-population correction. Notably, the trendlines for the WoE differences for the simple stratification and the STRmix^TM^ stratification are lower when $F_{ST}=0.01$ is used compared to when $F_{ST}=0$ is used, while the WoE difference for the full stratification method is shifted less. Using $F_{ST}=0.01$, the simple stratification method has a median WoE difference of −0.53 with a standard deviation of 0.57. The full stratification method is close to unbiased with a median WoE difference of −0.05 and a smaller standard deviation of 0.32. For the STRmix^TM^ stratification method the median WoE difference is −0.07 and the standard deviation is 0.72. The fraction of POIs for which the WoE difference is positive (i.e., the method is anti-conservative) is 12.4% for the simple stratification method, 15.4% for the full stratification method and 46.6% for the STRmix^TM^ stratification method. Although these fractions are non-negligible, most of the positive WoE differences are small in magnitude. The fraction of POIs for which the WoE difference exceeds one ban is 0.6% for the simple stratification method, 0 for the full stratification method and 6.6% for the STRmix^TM^ stratification method.

### 3.3. Contributors from the Same Population

**Figure 4:**
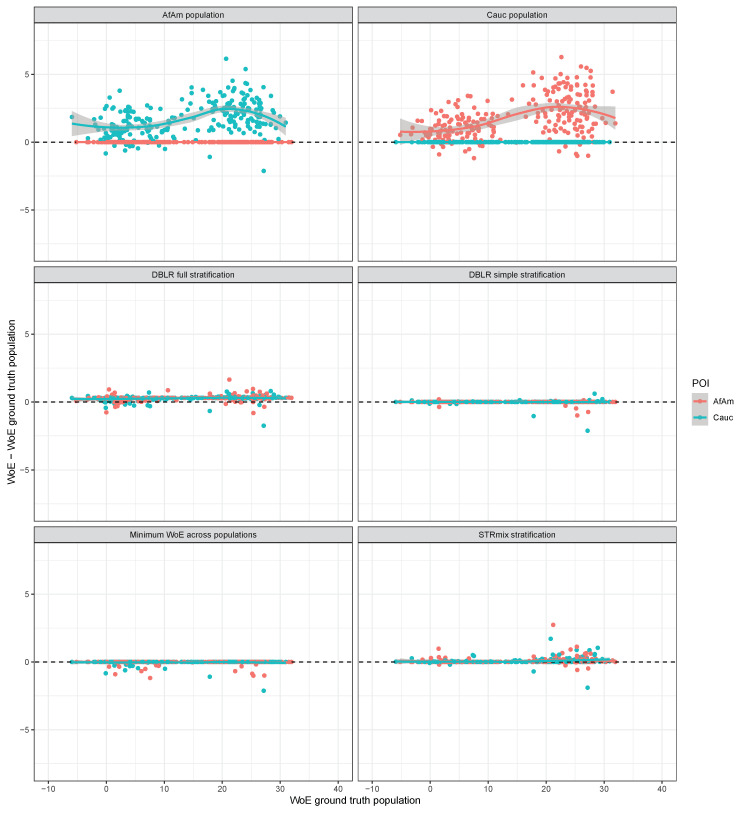
WoE difference between the compared methods and the WoE evaluated using the ground truth population with $F_{ST}=0.01$ for the 500 donors to 250 simulated two-person mixtures with contributors from the same population.

Part of the simulation study was repeated with both of the contributors in each of 250 simulated mixtures now originating from the same population. Figure 4 shows the likelihood ratios obtained for the 500 contributors when compared to the simulated mixtures using the six different likelihood ratio approaches. As before, the top panels show that using the wrong allele frequencies is non-conservative for almost all POIs. Interestingly, the full stratification method is slightly non-conservative in comparison to the ground truth population. For 94.6% of the POIs, the WoE difference is greater than 0 and the median difference is 0.30 bans (close to a factor of 2 in likelihood ratios). The factor of 2 appears because both under $H_{1}$ and $H_{2}$ the single large likelihood is the one with all persons coming from the same population, while under $H_{1}$ there are four permutations in total compared to eight under $H_{2}$. Alternatively, this can be seen as follows. Recall from the example calculations that we may write

$$\begin{array}{cc} {\mathrm{LR}}_{fully-stratified}= & \frac{P(O|H_{1},G_{p})}{P(O|H_{2},G_{p})}=\frac{\frac{1}{2}\left(P\left(O|H_{11},G_{p}\right)+P\left(O|H_{12},G_{p}\right)\right)}{P(O|H_{2},G_{p})} \end{array}$$

Assuming two populations (A,B) are considered and the only large likelihoods are obtained for $E_{1}=E_{2}=A$, we obtain

$$\begin{array}{cc} P(O|H_{11},G_{p})= & P\left(E_{1}=A,E_{2}=A|H_{11},G_{p}\right)P_{AA}\left(O|H_{11},G_{p}\right)+\\ \; & P\left(E_{1}=A,E_{2}=B|H_{11},G_{p}\right)P_{AB}\left(O|H_{11},G_{p}\right)+\\ \; & P\left(E_{1}=B,E_{2}=A|H_{11},G_{p}\right)P_{BA}\left(O|H_{11},G_{p}\right)+\\ \; & P\left(E_{1}=B,E_{2}=B|H_{11},G_{p}\right)P_{BB}\left(O|H_{11},G_{p}\right)\\ = & p_{A|G_{p}}\pi_{A}P_{AA}\left(O|H_{11},G_{p}\right)+p_{A|G_{p}}\pi_{B}P_{AB}\left(O|H_{11},G_{p}\right)+\\ \; & p_{B|G_{p}}\pi_{A}P_{BA}\left(O|H_{11},G_{p}\right)+p_{B|G_{p}}\pi_{B}P_{BB}\left(O|H_{11},G_{p}\right)\\ \approx & \pi_{A}P_{AA}\left(O|H_{11},G_{p}\right) \end{array}$$

In the same way we obtain $P\left(O|H_{12},G_{p}\right)\approx \pi_{A}P_{AA}\left(O|H_{12},G_{p}\right)$ and $P\left(O|H_{2},G_{p}\right)\approx \pi_{A}\pi_{A}P_{AA}\left(O|H_{2},G_{p}\right)$. Combining these, we obtain an expression for the fully stratified likelihood ratio ${\mathrm{LR}}_{fully-stratified}\approx \frac{\pi_{A}\frac{1}{2}\left(P_{AA}\left(O|H_{11},G_{p}\right)+P_{AA}\left(O|H_{12},G_{p}\right)\right)}{\pi_{A}\pi_{A}P_{AA}\left(O|H_{2},G_{p}\right)}=\frac{{\mathrm{LR}}_{A}}{\pi_{A}}$. In our example, we have $\pi_{A}=\frac{1}{2}$ so we get ${\mathrm{LR}}_{fully-stratified}\approx 2{\mathrm{LR}}_{A}$. The simple stratification method gives likelihood ratios that are for most POIs very close to the likelihood ratio computed with the ground truth population. The minimum WoE across populations is for most POIs equal to the WoE evaluated using the ground truth population and is otherwise slightly conservative. Finally, the STRmix^TM^ stratification method yields results that are most of the time very close to the ground truth population likelihood ratios.

## 4. Discussion

Despite an apparent consensus in the literature [12,14] that likelihood ratios incorporating population stratification are an elegant solution, little has been published about how such likelihood ratios should be evaluated exactly and how stratification methods compare to other methods. The goal of the current work was to partially address the lack of understanding in this area by proposing explicit methods and evaluating those using a simulation study. Related to this problem, several articles have previously described the effects of population database choice on kinship likelihood ratios [13,20]. Although these publications show that there are meaningful differences depending on which population database is used, another recent publication demonstrates that a universal (worldwide) database can be a good choice in the context of missing persons identification [21]. In the context of random match probabilities for full single source DNA profiles, Ref. [22] presented results that support the dismissal of racial databases and the adoption of a race-neutral database. The aforementioned articles do not discuss the context of probabilistic genotyping. The behaviour of likelihood ratio methods is especially important in this context, because more sensitive multiplexes increase the number of low-level and mixed samples that are analysed. The weight of evidence for such samples may be low and sensitive to modelling assumptions.

We have proposed two new likelihood ratio methods for population stratification. The first method, called simple stratification, is based on the assumption that all persons in the evaluation are from the same population. The second method, called full stratification, considers all permutations of assignment of persons to populations. The behaviour of both methods was investigated using a simulation study and compared to a method that is currently implemented in the STRmix^TM^ software. In the absence of sub-population correction (i.e., when $F_{ST}=0$), the findings indicate that the full stratification method is most accurate when the mixture contributors originate from different populations. The standard deviation of the WoE difference between this method and the ground truth is also the smallest among the compared methods. The simple stratification method is on average slightly conservative, however the standard deviation of the WoE difference between the simple stratification method and the ground truth is comparatively large (0.57 versus 0.32 for the full stratification method). This means that the simple stratification method is more often slightly non-conservative. The highest WoE differences observed for the simple stratification method are about 2 bans. The stratification method implemented in STRmix^TM^ is on average slightly anti-conservative with the median WoE difference being 0.31 bans. The standard deviation of 0.82 is higher than the standard deviation obtained for the other two stratification methods. The highest WoE differences were close to four bans. Besides the stratification methods, the simulation study also investigated the possibility of taking the minimum WoE across the two populations that were considered. This is, on average, a conservative approach. However, it is important to note that this approach does not take into account that the mixture donors originate from different populations and is therefore not always conservative.

When $F_{ST}=0.01$ was used in the likelihood ratio calculations, all approaches behaved more conservatively in the sense that lower WoEs were obtained. The WoE evaluated using the ground truth populations is affected relatively weakly by changes in $F_{ST}$ because the sub-population correction only applies within a population and not across populations. This explains why using the POI’s population, or a minimum across populations, is more conservative in the simulations where $F_{ST}>0$.

The current work was limited in scope. We have restricted the simulation study to involve two-person mixtures only to keep the volume of results manageable. Another limitation of the work is that we have assumed that a person originates from a single population. A natural extension would be to consider that persons may have two parents from different populations.

Finally, we mention an implementation detail relevant to software implementation of the models discussed in the current work. As discussed previously [17], DNA mixture calculations typically consider a virtual so-called Q allele that represents any allele not observed in a sample. When a POI is compared to a mixture and one of the POI’s alleles is not observed in the profile, it is (almost) irrelevant for the evidential value calculation which allele this is exactly depending on the particular model that is used. Population stratification methods introduce a complication, because the POI’s alleles that are not shared with the mixture may be informative of the population from which the POI originates and may affect the likelihood. Moreover, alleles at loci that are not shared between the POI and the mixture may also affect the likelihood.

## 5. Conclusions

The current work establishes that the common practise of taking a minimum WoE across populations is not always conservative when $F_{ST}=0$. Population stratification methods may also be non-conservative in some cases. When $F_{ST}>0$ is used in the likelihood ratio calculations, as is recommended, all compared approaches become conservative on average to varying degrees.

## Figures and Tables

**Table 1:** Mixture deconvolution for the electropherogram shown in Figure 1.

Genotype Combination (*s*)	Weight ($w_{s}$)
(13/14, 11/11)	0.3
(11/14, 11/13)	0.27
(11/13, 11/14)	0.23
(11/11, 13/14)	0.2

**Table 2:** Allele frequencies used in likelihood ratio calculation for the electropherogram shown in Figure 1.

	Frequency in	Frequency in
Allele (*a*)	Population *A* ($f_{a}^{A}$)	Population *B* ($f_{a}^{B}$)
11	0.6	0.5
13	0.3	0.3
14	0.1	0.2

**Table 3:** Overview of likelihood ratios calculated for comparison of each of the 500 true donors to the 250 simulated two-person mixtures.

Population	Implementation	$F_{\mathrm{ST}}$
African American only	DBLR^TM^, STRmix^TM^	0, 0.01
Caucausian only	DBLR^TM^, STRmix^TM^	0, 0.01
Simple stratified	DBLR^TM^	0, 0.01
Fully stratified	DBLR^TM^	0, 0.01
STRmix^TM^ stratified	STRmix^TM^	0, 0.01
Ground truth (mixed)	DBLR^TM^	0, 0.01

## Data Availability

Not applicable.
